# Navigating market access after conditional reimbursement: a communication roadmap for disinvesting orphan drugs

**DOI:** 10.1017/S0266462326103444

**Published:** 2026-01-16

**Authors:** Khadidja Abdallah, Alessandra Blonda, Brian Godman, Isabelle Huys, Kathleen Claes, Yvonne Denier, Steven Simoens

**Affiliations:** 1 Pharmaceutical and Pharmacological Sciences, KU Leuven, Belgium; 2 Strathclyde Institute of Pharmacy and Biomedical Sciences, University of Strathclyde, United Kingdom; 3 Public Health Pharmacy and Management, Sefako Makgatho Health Sciences University, South Africa; 4 Microbiology, Immunology and Transplantation, KU Leuven, Belgium; 5 Nephrology, Dialysis and transplantation, UZ Leuven, Belgium; 6 Public Health and Primary Care, KU Leuven, Belgium

**Keywords:** orphan drug, rare disease, disinvestment, resource allocation, health technology reassessment

## Abstract

**Objectives:**

Disinvesting from rare disease therapies with persistent clinical uncertainties following a managed entry agreement (MEA) may be necessary to ensure an equitable and sustainable healthcare system. To minimize public controversy, communication surrounding such disinvestment decisions must be timely, transparent, and tailored to stakeholder needs. This study develops a structured communication roadmap for disinvestment decisions in the Belgian context, addressing the clinical, financial, ethical, psychological, and social implications.

**Methods:**

Three advisory board meetings were conducted with nine experts from academia, clinical practice, health insurance funds, patient organizations, and the Belgian Drug Reimbursement Committee. A targeted review of peer-reviewed literature, legal texts, and policy documents informed the discussion guide. Meetings were transcribed verbatim and analyzed using NVivo 14, following grounded theory principles.

**Results:**

Disinvestment decisions should be evidence-based and communicated clearly and pragmatically in a context-specific manner. The proposed five-step roadmap defines the roles, responsibilities, and timelines of key stakeholders and provides guidance for public-facing documents, including a lay summary of the health technology reassessment report and written communications for patients and the public. Effective and timely communication with patients requires close collaboration among stakeholders. Moreover, regular engagement with healthcare providers and patients throughout the MEA period can enhance understanding and acceptance of the final decision.

**Conclusions:**

Transparent, collaborative, and adaptable communication strategies can facilitate the implementation of disinvestment decisions and help maintain trust among patients and the public. Although developed for rare disease therapies in Belgium, the proposed principles and roadmap are applicable to disinvestment processes in other healthcare systems.

## Introduction

To ensure optimal utilization of the pharmaceutical budget amidst constrained healthcare resources, monitoring the effectiveness and performance of commercialized drugs is imperative. This is particularly relevant for rare disease therapies that have the potential to improve morbidity and mortality but often enter the market with high costs and limited clinical evidence. This is usually due to small patient populations, disease heterogeneity, and a lack of adequate alternative treatment (1–5).

Box 1. Overview of the procedure for the HTA/reimbursement of rare disease therapies in Belgium.

In Belgium, a decision on the reimbursement of a health technology must be reached within 180 days following a manufacturer’s application (6). Throughout this process, technical experts from the National Institute for Health and Disability Insurance (NIHDI) prepare health technology assessment (HTA) reports at key milestones (day 60, day 90, day 120, and the final proposal on day 150) based on the manufacturer’s dossier and the conclusions of the Drug Reimbursement Committee (Commissie Tegemoetkoming Geneesmiddelen/Commission de Remboursement des Médicaments [CTG/CRM]). These reports should be publicly accessible along with a letter detailing the Minister of Health’s final decision, to ensure transparency of the process (7;8).

To address affordability concerns and mitigate the financial risk of reimbursing high-priced therapies with uncertain benefits, many health systems – including Belgium – increasingly employ managed entry agreements (MEAs). MEAs enable temporary and conditional reimbursement, coupled with confidential discounts and requirements for additional clinical or real-world evidence (8;9). However, their use has raised concerns. Several studies have highlighted that MEAs often fail to meet their contract terms, leaving clinical uncertainties unresolved and price negotiations incomplete (10–14).

In Belgium, MEA use has expanded rapidly, increasing from 13 new MEAs concluded in 2013 to 43 new MEAs concluded in 2023, with a cumulative total of over 183 MEAs established between 2013 and 2023 (15). MEAs are typically introduced when the Belgian Drug Reimbursement Committee (Commissie Tegemoetkoming Geneesmiddelen/Commission de Remboursement des Médicaments [CTG/CRG]) cannot reach consensus on reimbursement conditions at the end of the appraisal process (see Box 1).

In such cases, the applicant negotiates confidentially with the Minister of Social Affairs and Public Health to define the terms of the MEA. These agreements usually last up to 3 years, after which the agreement is either extended or a health technology reassessment (HTR) is performed. If evidence remains insufficient to justify continued reimbursement at the negotiated price, decision-makers may negotiate a new agreement – for example, including larger (confidential) discounts on the list price or revising requirements for evidence generation. Alternatively, the CTG/CRM may recommend that the Ministry of Health (MoH) discontinue the conditional reimbursement through disinvestment (16;17). However, this option is less common due to the controversial nature of disinvesting in treatments for rare diseases, which are often associated with a large unmet need.

Disinvestment can be passive, for example, by restricting reimbursement to certain subgroups with demonstrated benefit (18). Examples include the implementation of subgroup restrictions for ineffective anti-Alzheimer medications in France and Belgium (19;20) and the restricted prescribing of duloxetine in Sweden due to concerns over its value in practice following the availability of generic venlafaxine (21). However, this approach may be less suitable for orphan drugs, as patients often have no effective alternatives. Another form of passive disinvestment is the so-called “extinction scenario,” in which current (subgroups of) patients are allowed to continue treatment, but no new patients are initiated. In Belgium, this approach was applied to decitabine for acute myeloid leukemia patients with 20–30 percent blasts (22).

An alternative strategy is active disinvestment (or delisting), which involves the complete withdrawal of reimbursement for a disease therapy, requiring patients to pay the full cost if they wish to continue treatment. In Belgium, passive approaches are more common for rare diseases, whereas active disinvestment is avoided due to the lack of alternatives and the emotive nature of rare diseases (23). A notable example occurred in the Netherlands, when the College of Health Insurers recommended removing agalsidase alfa and alglucosidase alfa – used to treat Fabry and Pompe disease, respectively – from the basic reimbursement package. Despite an estimated incremental cost-effectiveness ratio of €15 million for alglucosidase alfa, the recommendation faced strong resistance from patients and physicians, and disinvestment plans were ultimately halted (24–26).

Nonetheless, as healthcare systems in high-income countries face growing financial pressures – driven in part by aging populations – active disinvestment of orphan drugs may become inevitable. This is to ensure the efficient and equitable allocation of resources to treatments that provide demonstrable value.

Previous research highlights that effective communication with stakeholders – including healthcare providers, payers, patients, and industry – is critical for successful disinvestment (27;28). In Belgium, a structured communication procedure exists only for ultra-rare diseases reimbursed on a case-by-case basis (29). In these cases, an Orphan Drug College provides an opinion on the individual prescription and reimbursement of the orphan drug (30). This College is composed of physicians and specialists knowledgeable about a specific rare disease (treatable by the orphan drug) and advisory physicians who are members of the reimbursement committee (CTG/CRM) at the National Institute for Health and Disability Insurance (NIHDI). They first deliver their opinion to the advising physician of the health insurance fund, who then informs the concerned patient. For other rare disease therapies, communication is often delayed, informal, or incomplete, leading to confusion and, at times, public controversy.

To our knowledge, this study develops the first structured, context-specific communication roadmap for the disinvestment of rare disease therapies. This roadmap is intended for situations in which the payer determines that the added value of a therapy – compared to the standard of care – has not been sufficiently demonstrated (or is considered insufficient relative to its additional cost). It seeks to address the clinical, financial, ethical, psychological, and social dimensions of orphan drug disinvestment. Moreover, it provides practical tools to guide communication with all stakeholders, especially patients and the wider public. While rooted in the Belgian system, the proposed principles and tools are designed to be adaptable to other jurisdictions with similar challenges in orphan drug disinvestment.

## Methods

### Study design

This qualitative, exploratory study used stakeholder engagement to develop a structured communication roadmap for the disinvestment of rare disease therapies. We applied grounded theory – an inductive methodology that facilitates the creation of frameworks based on collected data (31) – to capture actionable insights from expert knowledge. Grounded theory was chosen for its suitability in generating practical guidance for complex and sensitive decision-making contexts, making it highly suited to the study’s objectives.

### Study conduct

An advisory board was convened to guide the roadmap’s development. To prepare for the board meetings, we conducted a targeted literature review of disinvestment cases and strategies in healthcare, with a focus on rare disease therapies. Relevant peer-reviewed studies, legal and policy texts, and gray literature were identified through targeted searches in PubMed, Google Scholar, and snowballing. Search terms included synonyms for “disinvestment,” “delisting,” “discontinuation,” “reassessment,” and “defunding.” While not systematic, this review provided essential foundations to inform the discussion points for the meetings.

**Figure 1.**
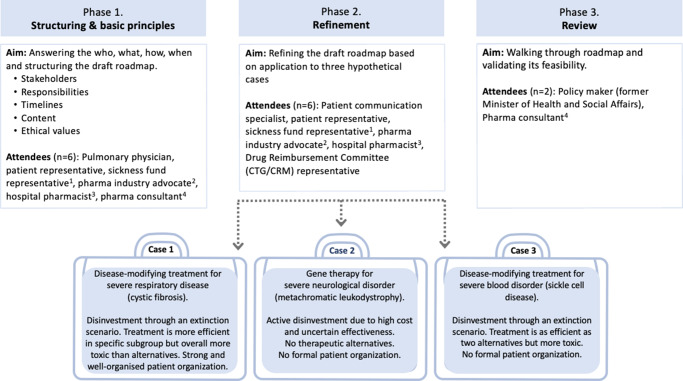
Overview of the roadmap development process and the scope of each meeting. Numbers in superscript indicate that the same expert participated in multiple meetings.

Three advisory board meetings (in Dutch) were held on 14 December 2022 and 22 and 30 May 2023. Feedback and insights from each meeting were integrated into subsequent discussions. The first meeting took place in person at the KU Leuven Department of Pharmaceutical and Pharmacological Sciences. The final two meetings were teleconferences to accommodate experts’ schedules. Figure 1 outlines the roadmap development process and the scope of each meeting.

The first meeting identified critical elements and values for effectively communicating disinvestment decisions to rare disease patients. Moreover, it determined which stakeholders should be involved, and at which stages, in developing and disseminating communication materials. Building on the results of this first meeting, a preliminary set of recommendations and a communication flow were developed and shared with participants ahead of the second advisory board meeting.

The second meeting produced a preliminary roadmap that was tested in three fictional cases. The third meeting validated the roadmap’s feasibility and practicality by discussing it in the context of two additional cases.

Based on these discussions, we created a five-step communication roadmap with supportive communication materials (see Supplementary Tables S1–S3), which include:

1. **A template for the lay summary of the HTR** report (Supplementary Table S2), modeled after the health technology assessment (HTA) reports from the Dutch Zorginstituut Nederland (ZIN) (32), the National Institute for Health and Clinical Excellence (NICE) (33), and the German Institut für Qualität und Wirtschaftlichkeit im Gesundheitswesen (34).
2. **A template for letters communicating** the disinvestment decision to patients and the wider public (Supplementary Table S3). It defines components to be included during, for example, a patient consultation, a press release, or the website of the national health authority.
3. **A roles and responsibilities form** for defining deadlines and stakeholders’ responsibilities (identify, inform, communicate, and publish; see Supplementary Table S3).

### Participants

We purposively sampled experts with experience in the Belgian (orphan) drug reimbursement process (35). Potential participants were identified through the investigators’ professional networks, scientific publications, and official websites or social media (e.g., LinkedIn) of national healthcare authorities. Experts were invited via email between 15 October and 30 November 2022.

The final advisory board included representatives from academia, health insurance funds, clinical practice, and the CTG/CRM. Recruitment ensured at least one representative from each stakeholder group and geographic diversity across Belgium.

### Data analysis

The focus groups were moderated by the principal researchers and transcribed verbatim by KU Leuven-affiliated coworkers. We followed the Qualitative Analysis Guide of Leuven to guide the process of qualitative data analysis (36). The same researchers analyzed the transcripts independently, by performing a two-stage analysis.

In the coding stage, a predefined (complementary deductive) coding framework, based on the specific study topics, was assigned to various text fragments. Additionally, inductive open coding was applied for emerging themes such as ethical values and prerequisites for disinvestment communication. Coding was performed in NVivo 1.7 by both researchers independently.

In the synthesis stage, the researchers compared their analyses to develop an overarching code structure, which was used to detail the roadmap steps. The two principal researchers then drafted the roles and responsibilities guide, lay summary template, and communication letter template. The full research team reviewed and validated the roadmap, principles, guides, and recommendations.

### Ethical considerations

The research protocol was approved by the Ethical Committee Research University Hospitals Leuven (14/10/2022, S66927). All participants provided written informed consent. Participant anonymity was maintained unless explicit permission for attribution was given. Data were handled confidentially throughout the study.

## Results

### Participant characteristics

Nine experts (four females and five males) participated in the advisory board meetings, of whom four attended sessions twice (see Figure 1). All were Belgian senior-level professionals with >10 years of experience in their respective fields, spanning academia, health insurance funds, clinical practice, and prior roles at the NIHDI (including the CTG/CRM).

### Principles for a successful communication roadmap

During the meetings, experts identified several bottlenecks that could hinder a smooth implementation of a communication roadmap – such as media controversies and inconsistent messaging. These challenges were synthesized into key principles essential for ensuring feasibility and effectiveness (see Table 1).

**Table 1.** Key principles for an effective communication roadmap for the disinvestment of orphan drugs

**Principle 1. Manage expectations of patients early on**The physician should proactively inform the patient about the conditional availability of their treatment from the start. Because treatments for rare diseases often involve significant uncertainty and are initiated in highly vulnerable patient populations, including children, it is crucial that physicians manage the expectations of patients, their parents, and other relatives.
**Principle 2. Ensure that the disinvestment decision is founded on appropriate data** It is essential that the decision made is based on the correct data. This also implies that the conditions of the MEA are clear from the start. For instance, the (intermediate) outcomes that are collected within the MEA should be relevant for clinical practice.
**Principle 3. Foster an early dialogue between manufacturers and HTA agencies, including patients and clinicians** This will broaden the support from the involved parties if a health technology is ultimately defunded. Additionally, a feedback loop should be established at multiple stages throughout the MEA and following reassessments to ensure the timely communication of uncertainties related to the real-world effectiveness of the orphan drug. This way, potential alternatives in the event of delisting could be identified. This approach can help mitigate the impact of delisting decisions and foster cooperation from manufacturers in developing both the health technology reassessment summary and written communication for patients.
**Principle 4. Maximize transparency where possible** There needs to be sufficient transparency on the content of the MEA and the uncertainties that it aims to answer. It is necessary to share information on which (intermediate) clinical outcomes were reached. In addition, the reasons behind the decision need to be communicated clearly and effectively to all stakeholders.
**Principle 5. Collaborate to convey a thoughtful and unified message** Throughout the process, it is crucial to leverage stakeholders effectively, ensuring their involvement where they can provide the greatest value. For instance, strong patient organizations, if present, should contribute to drafting public summaries and patient communications. The expertise of expert centers specializing in rare diseases should also be maximized. These centers not only educate primary caregivers about care pathways but also enable efficient and timely communication. Their multidisciplinary nature facilitates streamlined coordination of downstream steps in the communication roadmap, such as the rapid identification of all patients affected by a decision. Moreover, having a multidisciplinary team – including clinicians, nurses, dieticians, and other professionals – ensures that messages can be conveyed to patients through various channels, fostering a sense of support and providing opportunities for clarification. Early alignment of all stakeholders is also essential. This can be achieved through the multistakeholder meeting and drafting of the summary of the health technology reassessment report (Step 2), ensuring a unified message when responding to questions from patients, the media, or the broader public. Such alignment helps to reduce the risk of controversy and ensures consistent communication.
**Principle 6. Prevent information leaks ahead of official patient communication** To minimize the risk of controversy, it is essential to take proactive measures to prevent leaks of information regarding the final decision to patients before the official communication by their treating physician. To this end, all stakeholders involved in the decision-making process, as well as participants in the multistakeholder meeting, should be required to sign a nondisclosure agreement.
**Principle 7. Maximize the efficiency of the steps** To further prevent leaks, it is crucial to minimize the number of steps in the communication roadmap. Additionally, only the most relevant stakeholders should be involved at each stage. For example, during the multistakeholder meeting (Step 2), participation should be limited to those essential for defining roles and responsibilities and developing the communication strategy. To facilitate efficient progression through the steps, online meetings should be prioritized whenever possible and held as planned even if all stakeholders are not able to attend. This avoids unnecessary delays while maintaining a streamlined approach.
**Principle 8. Consider a sufficient grace period in cases of delisting** In cases of complete delisting, implementing a grace period (of, e.g., ~1 year) can help mitigate potential controversies. This interval provides adequate time between the decision and its implementation, ensuring stakeholders and affected parties have sufficient opportunity to prepare for the transition and explore alternatives.
**Principle 9. Provide sufficient resources to implement the communication roadmap** The reimbursement agency must have adequate human and financial resources to manage workload, draft reports, and ensure timely execution of the communication roadmap. This may include employing an in-house communication expert or hiring an external consultant to oversee key tasks, such as drafting public summaries, patient communication materials, and finalizing roles and responsibilities.
**Principle 10. Tailor the roadmap to specific cases with special attention to vulnerable groups** The roadmap must provide sufficient flexibility to accommodate the heterogeneity of orphan drugs and patients (e.g., vulnerable groups such as children and those mentally impaired). This with respect to the content of the communication materials and responsibilities of involved stakeholders. As such, each case should be individually discussed and tailored to its unique needs.

Abbreviations: HTA, health technology assessment; MEA, managed entry agreement.

Currently, Belgium has no formal system for communicating the disinvestment of a rare disease therapy that is conditionally or formally reimbursed by the NIHDI. Experts highlighted that clinicians often learn about such decisions informally or through delayed communication from manufacturers. Decision letters from the Minister of Health may provide only a brief rationale, often without a clear explanation of the evidence base. In some cases, patients first learn of a drug’s unavailability from their pharmacist.

### The five-step communication roadmap

**Figure 2.**
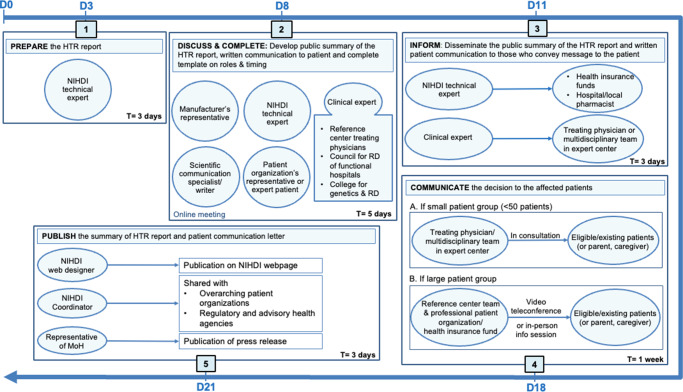
The five-step disinvestment communication roadmap. Abbreviations: D, day; HTR, health technology reassessment; MoH, Minister of Health; NIHDI, National Institute for Health and Disability Insurance; RD, rare disease; T, time.

The roadmap (see Figure 2) presumes that the competent authority or MoH formally communicates a decision to withdraw reimbursement to the manufacturer. The roadmap also presumes that the disinvestment decision is evidence-based and appropriate and, therefore, solely focuses on how the decision should be communicated. While the timelines are specific to Belgium, the structure can be adapted to healthcare contexts of other jurisdictions.

#### Step 1: prepare – compile the comprehensive HTR report

In Belgium, HTA reports are often available only upon formal request and may be difficult to interpret, even for those familiar with the process. The justifications provided in these reports are frequently unclear, lacking clarity in terms of language, scientific reasoning, and structure, which further complicates their accessibility and usability.

The first step is therefore to produce a publicly accessible HTR report that consolidates all relevant, nonconfidential evidence and clearly explains the reimbursement committee’s scientific conclusions. This report, prepared by NIHDI technical experts and tailored to the public, follows a format like the European Public Assessment Report of the European Medicines Agency (37), which avoids excessive technicality while maintaining sufficient detail. It aims to provide maximum clarity and transparency within the boundaries set by the confidentiality of CTG/CRM deliberations and price negotiations. Manufacturers may provide feedback on the draft. The compilation of this report should be completed within 3 days of initiating the procedure.

#### Step 2: discuss & complete – develop the public summary and communication guide

A NIHDI technical expert convenes a confidential multistakeholder meeting to prepare the communication to physicians, the patients, and the public. This step is best conducted as an online, rather than face-to-face, meeting to enable the earliest possible discussion. The goal of the meeting is to produce

- A public summary of the HTR report to the patient and public, including information on alternative treatment options (see guide for drafting the HTR report in Supplementary Table S1.
- A detailed communication letter for patients and the public (see guide for drafting communication letters in Supplementary Table S2) and
- A completed roles and responsibilities template, outlining actions, responsible stakeholders, and timelines for downstream communication and patient outreach (see template in Supplementary Table S3).

The NIHDI technical expert – who coordinates the meeting – invites participants via email or phone. All participants sign a nondisclosure agreement before the meeting to prevent premature leaks. The meeting requires the presence of various stakeholders, as detailed in Table 2.

**Table 2.** Stakeholders involved in drafting the disinvestment communication and their roles

Stakeholder	Responsibility	Additional notes
Government official	Presents the HTR report and explains the arguments behind the disinvestment decision to other stakeholders at the start of the meeting.	Could be the NIHDI technical expert responsible for the HTR report, along with a representative from the Minister of Health’s office in case the disinvestment decision deviates from the advice issued by the CTG/CRM of the NIHDI.
Manufacturer representative	Reviews and suggests changes to the public summary.	Participates in discussions to ensure accurate representation of the manufacturer’s position.
Clinical expert on the respective rare disease	Identifies the rare disease patients affected by the decision.	In Belgium, the expert is ideally from the rare disease reference center, the Council for Rare Diseases of Functional Hospitals, or the College of Genetics and Rare Diseases. These Centers, Councils, and Colleges are tasked with treating the patient group and, thus, can identify the rare disease patients to whom the decision needs to be communicated.
Patient representative or expert patient	Provides input on the format and content of the public summary and written communication for patients.	Identified by the clinical expert where relevant, or through established patient organizations, and offers a patient-focused perspective.
Scientific writer	Drafts the public summary and guide based on discussions during the meeting.	Can be an internal or external contractor of NIHDI.

Abbreviations: NIHDI, National Institute for Health and Disability Insurance; CTG/CRM, Commissie Tegemoetkoming Geneesmiddelen/Commission de Remboursement des Médicaments; HTR, health technology reassessment.

The duration of this meeting can range from an hour to half a day, depending on the complexity of the arguments behind the decision and the characteristics of the patient group. The scientific writer finalizes the public summary within 1 day after the meeting; feedback from attendees and approval from the MoH (or delegate, if the MoH was absent during the meeting) should be obtained within 3 days. Final documents are circulated within 5 days of the meeting.

#### Step 3: inform – prepare relevant stakeholders for patient contact

The public summary and communication letter are shared with physicians responsible for informing patients, as well as with stakeholders who may be approached by patients (e.g., health insurance funds and hospital pharmacists). The clinical expert (e.g., physician from the expert center, Council of Rare Diseases of Functional Hospitals, or College for Genetics and Rare Diseases), who was present in the virtual meeting, informs the multidisciplinary team of the reference center, or, where not applicable, the treating physician. Simultaneously, the NIHDI technical expert informs health insurance funds, the hospital, and/or local pharmacists. These parties should, above all, avoid confusion by providing appropriate and consistent messaging to patients or referring them to their treating physician. This step runs in parallel with Step 4 and should be completed within 3 days of finalizing the public summary.

#### Step 4: communicate – inform affected patients

Depending on the size of the target population, different actors should be involved in personally communicating the message to the concerned patient(s). In case of disinvestment in the form of a cohort restriction, both patients who are already receiving therapy as well as de novo or formerly eligible patients – who will no longer be able to initiate the treatment – should be informed. Patients already receiving therapy will be able to continue therapy, contingent on signing a form specifying that the treatment is no longer recommended. The message must be delivered empathetically by a trusted person, clearly stating that the treatment is no longer effective or beneficial. It should also reassure the patient (and their caregivers or relatives) by discussing alternative treatments and providing room for dialogue. Initially, this message should be conveyed verbally, followed by the delivery of the public summary of the HTR report (see supplemental guide).

For patient numbers below 50, the message can be personally delivered during a consultation with their treating physician (and the broader multidisciplinary team), who is responsible for personally reaching out and inviting the patients for a consultation. However, for patient numbers larger than 50, it becomes logistically challenging to communicate the message to everyone in a timely manner. In this instance, the multidisciplinary team of the reference center and the patient organization, if professional and well-organized, may invite concerned patients to an explanatory session. This session could be hosted on a video conferencing platform provided by NIHDI, or physically in the respective reference center. If there is no reference center or patient organization available, health insurance funds could alternatively organize such a session, in conjunction with advising physicians (and treating physicians). This session should conclude with a message urging patients to reach out to their treating physician for more specific and detailed information and refer to the HTR report and public summary. This step should occur simultaneously with step 3 and be completed within 1 week of receiving the written documents conveying the disinvestment decision.

#### Step 5: publish – release public statement and supporting documents

NIHDI publishes an official public statement, together with the HTR report, the public summary, and a communication letter to the patient, on an accessible and user-friendly NIHDI webpage. The official statement is concise, factual, and written in nontechnical language, specifying the decision and effective date, the principal reasons for disinvestment in lay terms, and links to the full HTR report and the public summary.

The webpage should be modeled after best-practice examples from the ZIN (The Netherlands) and NICE (The United Kingdom and Wales) (32;33). Simultaneously, the HTR report and letter to the patient are also shared with relevant patient organizations, national and regional agencies responsible national drug registration, pharmacovigilance and medical information provision – such as the Belgian Federal Agency for Medicines and Health Products and the Belgian Centre for Pharmacotherapeutic Information – and with other healthcare bodies likely to receive queries from clinicians or patients. A well-trained NIHDI spokesperson is appointed to answer any media questions regarding the decision, ensuring that all public messaging is consistent and accurate. Where possible, the timing of publication is coordinated so that patients receive the information directly from their treating physician prior to media reporting.

## Discussion

Several publications have described frameworks for prioritizing healthcare interventions or low-value care for disinvestment, de-adoption, or de-implementation (18;38–43). However, to our knowledge, this paper is the first to present a comprehensive roadmap specifically for communicating the disinvestment of rare disease therapies after conditional reimbursement. A particular strength of the roadmap lies in the practical guides and templates – such as the guide for drafting the public summary of the HTR report (see Supplementary Table S1), the guide for drafting the communication to patients and the public (see Supplementary Table S2), and the roles and responsibilities template (see Supplementary Table S3). These were developed bottom-up by a multistakeholder advisory board, which included patient representatives. While the timelines and specific stakeholders identified in the roadmap (e.g., the Orphan Drug College and the Council of Rare Diseases of Functional Hospitals) are a projection specific to the Belgian context, the general principles (Table 1) and supporting tools are transferable to other high-income countries with similar healthcare systems.

We also believe that our roadmap could be extended beyond pharmaceutical products to high-cost drugs or other health technologies. For example, considering new European Union quality control regulations, which are believed to be so stringent that they may prompt manufacturers to withdraw certain (orphan) medical devices from the market, our roadmap could provide guidance for decision-makers to inform patients about such market withdrawals (44;45).

### Principles underpinning a successful communication roadmap

Given the growing number of conditionally approved rare disease therapies and increasing concerns about the affordability of premium-priced biological medicines, there is a need for clear and transparent communication to patients and the public (13). This study identified 10 basic principles for successful communication of a disinvestment decision. These principles are likely to be applicable to the disinvestment of other health technologies in high-income settings, given the methodology used to develop them.

First, our findings showed that the disinvestment process and its rationale must be structured, evidence-based, and transparent. These characteristics are consistently identified in the literature as key facilitators of successful disinvestment strategies (18;27;38;39;42;46–52).

Second, fostering collaboration among key stakeholders – particularly clinicians and patients – is crucial for gaining acceptance of disinvestment decisions. Stakeholders should be engaged early and kept informed throughout the reassessment that precedes disinvestment (18;38;39;42;46;48;50;51). Patient engagement is essential to improve the acceptability of the decision (47;49). In the Belgian context, rare disease expertise centers play an essential role. Although there are currently eight such centers (53), they do not cover all rare diseases. Expanding their role and coverage, possibly by centralizing expertise per therapeutic area and formalizing responsibilities of key parties involved, could enhance the efficiency of communication and stakeholder engagement. Such approaches would facilitate timely inclusion of all relevant parties, reduce procedural delays, and minimize information leaks that could fuel public controversy.

Third, a communication roadmap should be practical, context-specific (46), and customized to the key parties affected by the decision (39;41). While we tested the feasibility and applicability of our roadmap in fictional cases, the heterogeneity of rare diseases requires flexible implementation of the roadmap and case-by-case adjustments. For example, the roles and responsibilities template (see Supplementary Table S3) should be expanded as needed to include parties essential for specific cases. Future adaptations of the roadmap should also incorporate tailored communication strategies for vulnerable populations, such as children and individuals with cognitive impairments. In such cases, communication might be directed first to their parents or primary caregivers.

Finally, a media response strategy should be built into the roadmap from the outset of conditional reimbursement. Highly publicized disinvestment cases – especially those involving treatments with no adequate alternatives – can generate strong media and public reactions. While healthcare payers typically appoint a spokesperson, our guides (see Supplementary Tables S1–S3) help ensure that the message is clear and transparent, and that all stakeholders are prepared to respond consistently to media queries.

### Limitations

This study has several limitations. We aimed to include experts from countries with relevant disinvestment experience and healthcare systems similar to Belgium. Of the 44 invited experts, eight were from France, six from the Netherlands, and four from the Walloon region. However, due to scheduling constraints in the post-pandemic period, only Belgian experts participated. Similarly, although we initially had commitments from five experts per advisory board meeting, last-minute cancellations resulted in smaller group discussions. Despite this, all major stakeholder groups were represented, and the risk of Flemish overrepresentation was mitigated by including experts active in national-level patient organizations and healthcare decision-making. Furthermore, while the roadmap was developed with consideration for vulnerable populations, time constraints during meetings prevented discussion of specific cases involving these groups. Given the exploratory nature of this study, future research should expand the advisory board to include a broader range of national and international experts and pilot the roadmap in real-world disinvestment cases.

## Conclusion

This study provides a comprehensive roadmap for communicating the withdrawal of funding for rare disease therapies after conditional reimbursement. It also identifies core principles essential for effective communication of disinvestment decisions. Although designed for the Belgian context, the roadmap’s principles and tools can be adapted to other jurisdictions. By applying evidence-based, transparent, collaborative, and adaptable approaches, policymakers can improve the communication of disinvestment decisions, ultimately benefiting patients, clinicians, and the public.

## Supporting information

10.1017/S0266462326103444.sm001 Abdallah et al. supplementary material Abdallah et al. supplementary material

## Supplementary material

The supplementary material for this article can be found at http://doi.org/10.1017/S0266462326103444.
