# Supplementary material for: Navigating market access after conditional reimbursement: a communication roadmap for disinvesting orphan drugs
Source: Int J Technol Assess Health Care. 2026 Jan 16;42(1):e15. doi: 10.1017/S0266462326103444 (PMC12916244; doi:10.1017/S0266462326103444)
Supplement: Abdallah et al. supplementary material [file S0266462326103444sup001.zip › Supplemental_Material_S2_251022_CLEAN.docx]

**Table S2.** Guide for drafting a communication letter for patients

| **The arguments behind the decision**  Briefly address the decision that has been made. Convey this message with empathy, providing an explanation of the main arguments for disinvestment. Focusing on the ‘why’ may limit the resistance by patients. Issues regarding safety and efficacy should be addressed. This enables conveying a positive message, namely that disinvestment is not a negative rationing approach but rather one that puts quality and safety of the patient first. |
| --- |
| **Alternative treatment(s)**  A section that focuses on how the patient will be supported after access to the treatment has been restricted. Explain that the patient will receive the best option available.  This means that all available alternatives should be addressed, including non-pharmaceutical options like physiotherapy or self-management. Focusing on alternatives provides a sense of reassurance for patients who may feel left behind by the disinvestment decision.  Explain which steps are taken in the meantime, between now and disinvestment, to help the patient transition to the best possible options. |
| **Reaching out to third parties**  A brief statement motivating the patient to contact their primary healthcare provider/clinician at the Rare Disease Reference Centre, in case they have any further questions regarding their therapy.  A brief statement motivating the patient to reach out to their sickness fund in case of further questions.  Provide the contact details of the concerned patient organisation(s). |
| **Legal statement**  To address legal requirements, it is essential to incorporate a statement that guides patients towards designated authorities as an option to contest the decision. |
