# Supplementary material for: Navigating market access after conditional reimbursement: a communication roadmap for disinvesting orphan drugs
Source: Int J Technol Assess Health Care. 2026 Jan 16;42(1):e15. doi: 10.1017/S0266462326103444 (PMC12916244; doi:10.1017/S0266462326103444)
Supplement: Abdallah et al. supplementary material [file S0266462326103444sup001.zip › Supplemental_Material_S3_251221_CLEAN.docx]

**Table S3.** Template outlining actions, responsibilities and timelines

|  | **Action** | **Responsible** | **Due date** |
| --- | --- | --- | --- |
| **Discuss & complete**  **(Step 2)** | If no patient organisation, identify the patients to join the virtual round table | [name] | [date] |
|  | If no reference centre: provide a list of primary/peripheral care providers | [name] | [date] |
| **Inform**  **(Step 3)** | Inform the multi-stakeholder team within the Reference Centre | [name] | [date] |
|  | Inform the primary/peripheral healthcare provider | [name] | [date] |
|  | Inform the sickness funds | [name] | [date] |
|  | Inform the hospital pharmacies | [name] | [date] |
| **Communicate**  **(Step 4)** | Provide a list of affected patients | [name] | [date] |
| **Publish**  **(Step 5)** | Release official public statement with links to supporting documents (HTR report, HTR summary to patients and public, communication letter to patients) | [name] | [date] |
|  | Publish statement on the websites of the sickness funds | [name] | [date] |
|  | Organise press release/webinar/Q&A session for patients | [name] | [date] |

*Abbreviations: HTR, health technology reassessment*
