# Supplementary material for: Navigating market access after conditional reimbursement: a communication roadmap for disinvesting orphan drugs
Source: Int J Technol Assess Health Care. 2026 Jan 16;42(1):e15. doi: 10.1017/S0266462326103444 (PMC12916244; doi:10.1017/S0266462326103444)
Supplement: Abdallah et al. supplementary material [file S0266462326103444sup001.zip › Supplemental_Material_S1_251022_CLEAN.docx]

**Table S1.** Guide for drafting the summary of the HTR report for patients and the public

| **Recommendations**  This section describes the final recommendation of the reimbursement committee (CTG/CRM). It details whether the MoH followed the decision of the reimbursement committee, and provides clear information on the change in the reimbursement condition |
| --- |
| **Main arguments behind the disinvestment decision**  This section briefly addresses the main reasons behind the disinvestment. Especially, in the case of safety, efficacy or effectiveness issues. It is important that these are explained in layman’s terms and prioritise the patient’s wellbeing. |
| **General information on the orphan drug**  This section provides general information on the orphan drug, such as:   - The active ingredient and strength - The commercial name - The dosage regimen - The route of administration - The public price |
| **Safety evaluation**  This section provides an overview of the most relevant points of the safety evaluation of the orphan drug. It includes an evaluation of the key safety outcomes. It provides a summary of the information as described in the HTR report. |
| **Efficacy evaluation**  This section provides an overview of the primary points of the efficacy evaluation of the orphan drug. It provides a summary of the information as described in the HTR report. |
| **Effectiveness - Real-world evidence assessment**  This section describes how real-world data was collected and assessed in the context of the conditions set forth by the MEA. Therefore, it should answer the following questions:   - Which uncertainties were to be answered by the MEA? - Which uncertainties were (un)resolved, why? - Which data was collected to answer each uncertainty? - Which steps were taken to address each uncertainty? - Were the outcomes, for which data had to be collected, realistic and representative of clinical practice? - Were patient-reported outcomes collected and assessed? If so, what were the results? |
| **Overview of the reassessment process**  This section provides a timeline of the whole process, including:   - The date of initial approval of the MEA - The date of submission of (real-world) data - The different points for reassessment - The date of the CTG/CRM vote - The final decision by the MoH |
| **Involved parties**  Provide a list of all parties that were involved and consulted at various points throughout the reassessment process |

*Abbreviations: CTG/CRM, Belgian Drug Reimbursement Committee; MoH, Minister of Health; MEA, managed entry agreement; HTR, health technology reassessment*
